# Donation Care Work: Family Members' Contributions to Deceased Organ Donation Following Determination of Death Using Circulatory Criteria

**DOI:** 10.1111/1467-9566.70257

**Published:** 2026-09-06

**Authors:** Amanda van Beinum, Vanessa Gruben, Aimee J. Sarti, Sonny Dhanani, Lindsey McKay, Jennifer A. Chandler

**Affiliations:** ^1^ Department of Sociology York University Toronto Ontario Canada; ^2^ Children's Hospital of Eastern Ontario Research Institute Ottawa Ontario Canada; ^3^ Faculty of Common Law University of Ottawa Ottawa Ontario Canada; ^4^ Department of Medicine University of Ottawa Roger‐Guindon Hall Ottawa Ontario Canada; ^5^ Department of Critical Care The Ottawa Hospital General Campus Ottawa Ontario Canada; ^6^ Pediatric Intensive Care Children's Hospital of Eastern Ontario Ottawa Ontario Canada; ^7^ Department of Environment, Culture and Society Thompson Rivers University Kamloops British Columbia Canada

**Keywords:** care work, death and dying, end‐of‐life care, families, organ donation

## Abstract

Family members of potential deceased organ donors are often portrayed as gatekeepers to the individual experience of organ donation. We argue the success of deceased donation as an institution depends on often‐unrecognised family labour that occurs after consent. Building on feminist notions of care work, we propose the concept of ‘donation care work’ to conceptualise the combination of emotion work, end‐of‐life care, and death work that family members perform to support efforts for donation while facing imminent loss. We interviewed 38 family members of potential deceased organ donors in Canada. Family member narratives revealed ongoing contributions throughout donation attempts, including managing uncertainty and reconciling difficult emotions before, during and after the donation attempt. Understanding family contributions to deceased donation as work introduces capacity for labour as a contributing factor potentially impacting willingness to consent. The concept of donation care work furthers the sociology of donation by offering a framework to map the essential efforts of family members and the institutional reliance on their labour, even when donation attempts do not lead to transplantable organs.

## 1 Introduction

This paper responds to Dimond et al.'s (2019) call for the development of a sociological theory of donation. Building on the work of others who have proposed the need to reconfigure the concept of donation (Łuków 2020; R. M. Shaw 2015; Zeiler 2014), we focus on the role of close friends and family (hereafter referred to as family members) as an integral part of the process. Specifically, we develop in this paper an analysis of the role of family members as extending beyond consent for donation. Drawing on family members' experiences with attempts for organ donation after death determination using circulatory criteria (DCC), we outline how donation is made possible in part through families' active contributions. We develop the concept of ‘donation care work’—the affective and physical labour of caring for the dying while preserving the possibility of donation—as a framework to make visible the work of family members within the collective social project of deceased donation.

Deceased organ donation following DCC involves removal of organs from patients determined dead after blood circulation ceases (a different pathway than ‘brain dead’ donation). Family members are asked to make decisions about withdrawal of life sustaining therapies,^1^ to consent to organ donation and to assist with screening questionnaires^2^ prior to the determination of death. Families who agree to donation must wait for eligibility testing and retrieval team preparedness before removal of the ventilator can take place. For donation to proceed, potential DCC donors must be determined dead within specific time windows following ventilator removal (∼30 minutes–3 hours), before organs are damaged by lack of oxygen (Kalisvaart et al. 2021). Up to a third of attempts do not result in organ procurement due to the length of the dying period (Suntharalingam et al. 2009). Deceased donation after DCC modifies the cadence and content of end‐of‐life care, with implications for family member experiences. Our study was designed to explore how family members experienced intersections between end‐of‐life care and donation after DCC, generating knowledge about the role of families in upholding the institution of deceased donation.

## 2 Background

The donation of human body parts relies on systems of exchange commonly theorized as a gift relationship motivated by altruism (Machin et al. 2020). Deceased organ donation is often referred to as the ‘gift of life’ due to the life‐saving nature of organ transplantation. This framing positions donors as selfless heroes acting for the common good, despite the reality that deceased donation decisions are often left for family members because potential donors are likely to be unconscious (Delgado et al. 2019). Healthcare providers in many jurisdictions ask permission from family members prior to donation (even if a patient's wishes are known) despite arguments that this contravenes legal principles of consent (D. Shaw et al. 2017).

Theoretical analyses of the gift narrative often assume that the interests of potential donors and family members are interchangeable—that both share similar altruistic motivations for donating organs (Łuków 2020). Hesitations about donation are often attributed to a lack of knowledge about donation or different cultural beliefs. These assumptions obscure the realities of unexpected death and bereavement facing family members and do not account for the fact that families make decisions about consent in different contexts than when people register their intent to donate.

Some scholars have suggested that instead of a gift of life, deceased organ donation is better theorized as a sacrifice. The same Christian mythologies that feed into the idea of organs as gifts contribute to the idea of donation as a self‐sacrifice in which something essential to the self is given up for the sake of the other (Mongoven 2003; Parsons et al. 1978). Although donors literally sacrifice their bodies, family members who agree to donation sacrifice control over aspects of the process of dying and access to the dead body (Sque et al. 2006). Although families rarely have complete control over the dying process in intensive care, the addition of an attempt for donation may further reduce possibilities for choice due to requirements for organ procurement. The framework of sacrifice draws attention to how perseverance through pain and difficulty make donation a meaningful contribution. Sque et al. (2006) have suggested that understanding which narratives (e.g., gift of life or sacrifice) become most meaningful for families could improve consent discussions.

For many families, and especially in the contexts of deceased donation in which consent for donation occurs prior to death, consent marks the starting point of activities establishing conditions for organ procurement to proceed. Bea (2020) has described donation after DCC as an assemblage of relational and material components which come together to make organ procurement possible. Healthcare workers orchestrate many of these activities, including the work of ‘death brokering’ in which end‐of‐life care of potential donors is made to seem ‘routine’ (i.e., adhering to usual practice) despite constraints such as having to be in or near an operating room (Cooper and Murphy 2025). Understanding donation after DCC as an assemblage draws attention to institutional and system‐wide factors contributing to its success, over and beyond altruistic motivations of individual donors (Bea 2020).

The role of family members within this assemblage of donation after DCC has been undertheorized. Existing research studies suggest family members of potential DCC donors are likely to remain present at the patient's bedside (Prescott et al. 2019). Donor family members have identified involvement in the end of life and processes of donation after DCC as important components of their experiences, especially when they feel adequately prepared (Renet et al. 2026; Sarti et al. 2025). Family members of people who do not die in time to donate may also experience a ‘second loss’ of unsuccessful donation (Taylor et al. 2017), and a ‘triple loss’ when they perceive negative impacts on waiting recipients (Sarti et al. 2025). There is a need for analytical frameworks to make sense of these concerns to better understand how family members manage the disrupted death and dying experiences resulting from deceased donation after DCC (Rogers 2022).

In this paper, we use the analytical lens of care work to make sense of the role of donor family members beyond the moment of consent. Analysing family member involvement as work draws attention to how this often unrecognised labour plays a role in sustaining the social institution of deceased organ donation.

### 2.1 Donation Care as Work

The concept of care work originates from feminist political economy analyses of social reproduction (e.g., the work of creating and maintaining a labour force). The affective and material labour of social reproduction is known as care work (Black 2020). This includes the tasks needed to sustain and reproduce life: providing direct physical and emotional support, fostering relationships and maintaining environments adequate to living (Klostermann 2025). Care work is also essential for supporting and maintaining social institutions (Ahmed 2021). The act of caring has traditionally been understood as a ‘natural’ activity performed mainly by women. As a result, caring has been taken for granted, undervalued, and relegated to low‐status individuals (Luxton 1980). Framing acts of care as work pushes back against its devaluation and facilitates recognition of how care is both essential and unevenly allocated (Braedley and Luxton 2021).

At least four existing sub‐categories of care work are relevant to deceased donation, yet none capture the specific labour of caring for life‐in‐death which is entailed in the contexts of donation after DCC. We outline these categories in more detail below before proposing a new concept of donation care work to describe the work families do to sustain the project of deceased donation after DCC.

The first related sub‐category is emotion work, an integral part of care work. The work of managing emotions helps maintain adequate social environments. People manage emotions to make others comfortable and to match social expectations or ‘feeling rules’ operating in particular contexts (Hochschild 1979). Attending a dying person's bedside, for example, may carry expectations about appropriate emotions, with some families insisting on maintaining a positive affect while others expect a sombre mood. Family members perform emotion work when they manage affect to meet these expectations, work which may involve suppressing contravening emotions and seeking alternative spaces to process inner conflict (Wilkinson and Wilkinson 2020). Emotion work at the end of life can become exhausting as family members remain continuously present at the patient's bedside (Wilkinson and Wilkinson 2020). Emotion work in the contexts of donation attempts involves management of emotional friction as families cycle between grief and hopefulness.

A second related category exists within the concept of end‐of‐life‐care. End‐of‐life care involves the work of responding to the vulnerabilities of the dying. In addition to emotion work, a large component of end‐of‐life care involves what Wilkinson and Wilkinson (2020) call ‘dignity work’ of dealing with physical realities of failing bodies. In the hospital contexts of deceased donation, much of the physical work of end‐of‐life care is handled by healthcare professionals who maintain the patient's body and manage life support machines. Family members may perform some direct care, for example through assisting with grooming. They also bear witness to the realities of failing bodies when present for procedures such as removal of the breathing tube. Family members perform important social roles of accompanying the dying through the social transition of death. Symbolic practices like the bedside vigil help to maintain social cohesion through this period of disruption (Kellehear 2013). The framework of care work helps to draw attention to these activities of end‐of‐life care as labour—active and meaningful contributions that family members make time for when attending their dying loved ones.

Death care is a third category of care work related to deceased organ donation. Death care includes the work of dealing with dead bodies, such as making funeral arrangements, organising transport, and preparing ceremonial disposal (Collin 2026). In addition to physical and administrative labour, death care also contributes to emotional burden. How people interact with bodies after death, including through funeral practices, has a strong influence on trajectories of grief (Berthod et al. 2024). The contexts of deceased donation after DCC require donors to be removed to the operating room immediately after death, limiting time with the body. Successful donation may also offer a narrative that helps to transform the tone of grief work. Family members of potential donors may plan for and execute different versions of death care depending on whether donation proceeds.

Finally, in addition to emotion work, end‐of‐life care, and death care, family members of potential donors are also engaged in donor care, a category describing the work of sustaining the possibility of organ donation. For family members of people who become donors after brain death, donor care includes the work of managing cognitive dissonance resulting from acknowledging death while experiencing signs of life from the body (Sque and Payne 1996). In contrast, DCC donors only acquire the label of ‘donor’ once death has occurred within required timelines and organs are retrieved. Therefore, family members mostly participate in caring for *potential* donors. A new concept is needed to describe the work performed by families during contexts of donation after DCC: caring for the dying while preserving the possibility for organ donation.

We propose the concept of donation care work to describe the contributions families make to the deceased donation process, regardless of whether organ procurement occurs. Donation care work involves performance of end‐of‐life care and death care while conducting the emotion work required to manage the potential prospect of deceased donation alongside the grief of impeding death. It involves meeting expectations for a ‘good death’ (i.e., without pain or suffering) while preserving the possibility for donation. In some cases, it includes dealing with disappointment that despite all efforts, donation was not possible. Performing donation care work is often meaningful for family members, despite its difficulties. We argue that donation care work provides a concept to make visible the important and overlooked contributions family members make to the institution of deceased donation. Using the concept of donation care work allows us to analyse family contributions as meaningful labour rather than ‘natural’ responses. It also facilitates analyses of inequalities. Finally, it provides a conceptual framework to expand the study of donor family members beyond their involvement in consent authorisation.

## 3 Methods

This qualitative study involved interviews with close friends and family members (hereafter collectively referred to as ‘family members’) of people eligible to donate organs after DCC. We invited anyone involved in the decision‐making process to participate, independent of legal decision‐making status. Family members of potential donors were enroled in conjunction with a study exploring the physiology of dying after the removal of life sustaining therapies in 20 ICUs across 3 countries (Dhanani et al. 2021). Recruitment into the present study took place in a subset of 8 ICUs in two Canadian provinces. Enrolment of family members was conducted by a local research nurse at each site. After family members had participated in discussions about organ donation after DCC with an organ donation coordinator, an experienced research nurse approached to ask if they were interested in a follow up phone call about an interview‐based research study. We contacted family members by phone 6–18 months after the patients' death to explain the interview study and ask for their consent. This period of delay in re‐contact is within the range of comparable studies of deceased donor family experiences (Hoover et al. 2014; Sarti et al. 2022). Many family members later reflected how contributing to research during the peri‐mortem period had been a positive experience (van Beinum et al. 2021).

A retrospective enrolment strategy was later facilitated through partnership with an organ donation organisation (ODO).

Family members who consented to participate completed a 45–60 minute semi‐structured interview by phone or in person at the participant's home with Anon, Anon, Anon, or Anon, all experienced qualitative researchers.

Interviews encouraged participants to discuss their experiences in the ICU, including with decision making, end of life care processes, and experiences post‐death (see Interview Guide, Supporting Information S1: Appendix A). This study was approved by the research ethics boards of the university (File no. 04‐15‐25, File no. 04‐15‐25B), all seven participating hospitals, and the ODO.

All interviews were audio recorded and transcribed by a professional transcription service. We used Atlas.ti (ATLAS.ti Scientific Software Development GmbH 2019) to organise qualitative analysis.

A three‐part analysis was conducted using the approach of inductive content analysis. This approach involves the classification of textual data through coding and identifying themes or patterns, involving a systematic orientation to subjective interpretation (Elo and Kyngäs 2008). Phase 1 involved constant comparative analysis; data were continually revisited and compared to other pieces of data in an iterative process (Guest et al. 2014). A.V.B, V.G., J.A.C. and A.J.S. read and coded the first five transcripts, meeting after each to discuss key ideas and identify emerging codes. Initial codes were organised into broad categories of either chronological or overarching codes to differentiate between moments of time in participant stories and central themes which emerged throughout. Emerging codes were compared during review meetings to identify similarities and differences and refine definitions. Differences between coders were discussed and we produced a draft coding guide. A.V.B., V.G., J.A.C. and A.J.S. then coded two transcripts together, revising and refining the coding guide as needed until we produced a final code book. Phase 2 involved consistent coding of all transcripts. A.V.B., V.G., J.A.C. and A.J.S. each independently read and coded subsets of 10 transcripts, with each transcript coded by at least two authors. A.V.B. read through all coded transcripts to ensure coding consistency. Discrepancies were resolved at regular meetings. In Phase 3 we were guided by processes of subjective interpretation and narrative building (Braun 2022). Once all transcripts were consistently coded, A.V.B., V.G. and J.A.C. read through all interview segments associated with specific codes to extract sub‐themes for further analysis. We met regularly to discuss emerging trends and identify sub‐themes for final analysis and presentation.

Our interdisciplinary research team brought a wealth of prior experience and a diversity of professional approaches to analysis. Our team includes expertise in medical sociology (A.V.B. and L.M.), law (V.G. and J.A.C.) and critical care medicine (A.J.S. and S.D.). Several authors have been involved in the development of practice guidelines for donation and transplantation (S.D., J.A.C. and A.J.S), and J.A.C. has held formal roles in the Canadian Society of Transplantation. Each team member contributed different perspectives to thinking about the role of families within the process of donation after DCC which at times led to the emergence of conflicts and contradictions. Our analytic process is guided by the interpretation of content and ultimately aims to remain close to what families told us during interviews. Coding and analysis were conducted iteratively to surface and resolve differences in interpretation, rather than imposing a single disciplinary perspective.

Due to the richness of the interview data, it was not possible to present all findings in a single manuscript. In this paper, we present themes relating to family members' experiences of end‐of‐life care and the outcomes of donation after DCC, findings which contributed to our development of the concept of donation care work.

### 3.1 Study Limitations

Our interviews with family members of potential and actual deceased organ donors cared for in a small subset of Canadian ICUs are not representative. The addition of recruitment through an ODO may have increased likelihood of participants strongly committed to organ donation. We did not collect demographic characteristics such as race and income and thus were unable to apply an intersectional approach. We were able to reach saturation in our sample and found a high degree of repetition of core themes across participant narratives. Although these stories are not representative, they provide an important contribution to practical and theoretical conversations about organ donation after DCC.

## 4 Results

We recruited 33 family members of 28 patients through a prospective clinical cohort study and 10 family members of 9 patients through a retrospective strategy. Interviews were conducted a median of 9 months after death (8 months for prospective, 13 months for retrospective enrolment). Family members of 32 patients, which included 38 individual participants (in some cases, more than one family member participated in the study) completed 35 interviews (some family members participated in interviews together) between September 2016 and June 2018. For this analysis, five interviews were excluded (4 families who declined consent, 1 family whose loved one became a brain‐dead donor). All names used in this manuscript are pseudonyms.

### 4.1 Participant and Patient Demographics

The majority (29/38, 76%) of family members were female and cared for male patients. Fourteen family members attended a patient from whom organs were procured after DCC (OP), 17 attended a patient from whom organs were not procured (NP) and 4 attended a patient who was deemed ineligible for donation during testing (IE). The most common relationship to the deceased was as a partner (12/38, 32%) or parent (12/38, 32%). Patients' average age was 42 years at time of death (range: 18–69 years). Patients for whom organs were not procured did not die on time to contribute solid organs, though many donated tissues (e.g., corneas, skin).

### 4.2 Reconciling Deceased Donation and Technological Dying in the ICU

Three significant periods emerged within the process of potential donors' dying in the ICU: (1) the period of waiting between decision to remove the ventilator and the start of the procedure, (2) moments of being with the patient while life supporting technologies were removed and (3) the period of waiting for death to occur so organ donation could take place. Across all three, many participants described how they managed emotions and took on various activities they felt were expected as part of attending the dying.

#### 4.2.1 From Decision to Removal: ‘More Time’ Versus ‘Fake Time’

Almost all families described having to wait between their decision to remove life sustaining therapies and when ventilator removal began. About half of participants experienced this delay as positive because it allowed for additional time with the patient. They described the benefits of having ‘more time to be around each other’ (Donna, spouse, NP), as time for saying goodbye. For these families, delays in removing the ventilator facilitated the production of a socially recognisable period of dying and thus a space in which important end‐of‐life rituals like the bedside vigil could take place. Vigils for the dying are an enduring end‐of‐life tradition (Kellehear 2013). They establish a time for families to come to terms with imminent death and to begin constructing narratives of hope and continuity for life beyond death. For some study participants, the decision to remove life sustaining therapies appeared to initiate the beginning of the social transition of dying. Delays in removing the ventilator thus made space for ‘more time’ with the dying to make sense of impending death and imagine future hopes, including the hope for successful organ donation.

For the remaining participants, delays were experienced as challenging. Family members recounted being exhausted after days of making life and death decisions, which made unforeseen waiting time feel additionally burdensome. Some participants expressed concern that additional hours or days on life sustaining therapies might cause the patient further suffering.

One aspect of difficulty was contending with the knowledge of the patient's impending death, whilst unable to begin grieving the loss:

… it was just more time with him, but it was like fake time, I think, at a certain point we were just looking forward for closure.

(Wren, sibling, NP)

In some cases, participants described delays spanning hours in which families remained ‘in limbo’ (Casey, sibling, NP), believing each subsequent hour would bring the time for ventilator removal. After experiencing repeated delays, Diane, sister to a potential DCC donor, described threatening to withdraw consent for organ donation unless a clear timeline for ventilator removal could be confirmed.

For these family members, delay prolonged experience in an uncomfortable liminal state of anticipation. Instead of an intrinsically meaningful social transition, extended dying was experienced as a barrier to desired outcomes. This finding aligns with Selter et al.'s (2022) observations of trends in Western cultural contexts towards a preference for rapid and painless deaths rather than a gradual decline in which to say goodbye. When participants in our study had already said their goodbyes, delaying ventilator removal created unwanted extensions in the dying process and resulted in the production of ‘fake time’ (Wren, sibling, NP) which had to be endured to get to the desired endpoint of closure.

Brenda's (spouse, OP) comments illustrate the challenges of an extended end‐of‐life trajectory involved in donation after DCC:

… we were ready to let [the patient] go and be at peace and then we had to wait 24 hours, that was the hardest part. […] it was mixed, you know, because we knew we had another day with him. I'm sorry [crying] …I knew we had another 24 hours with him so, you know, that was good. And to know that this is what he wanted and there was a good possibility he would be able to help other people was—I was okay with that, with the decision of waiting.

(Brenda, spouse, OP)

Brenda describes an additional day of waiting for life support to be removed as ‘the hardest part' of her experience. Waiting entailed not being at peace and not being able to make the social transition to bereaved partner, even though she had already accepted the inevitability of his death. Brenda describes how the possibility of achieving a successful donation and alignment with her partner's wishes helped to provide justification for enduring this difficult waiting period. Nevertheless, her tears underscore the significance of this waiting period as emotionally challenging. Family members' endurance of waiting between the decision for ventilator removal and initiation of withdrawal of life support was work. It involved management of difficult emotions and maintaining physical presence through the caring activities of the bedside vigil.

#### 4.2.2 Presence During Dying: ‘Being There’ Versus ‘Not Wanting to See’

Another theme concerned participants' experiences of being present at the bedside during the dying period. Just under half of all family members described staying with patients for all or part of the process of withdrawal of life sustaining therapies.

Participants described not wanting to leave patients alone during their final moments, and to retain a form of caring presence such as holding the patient's hand. The ability to be physically close to the patient and to talk to them during their final moments was important, and family members felt grateful they had been able to remain nearby.

However, remaining present was a difficult experience. Four family members described leaving the room during distressing procedures such as removal of the breathing tube. Participants described ‘staying as much as they could’ (Logan, sibling, NP) but mentioned how remaining at the bedside for hours and sometimes days following ventilator removal was emotionally and physically exhausting.

A few participants chose not to be present at the hospital for the removal of life support. They justified the decision due to overall exhaustion, having ‘already said [their] goodbyes’, (Terry, parent, OP) and not wanting to witness potentially distressing final moments. In some cases, participants explained how friends, family members, or healthcare professionals stepped in to perform the role of ‘being there’. Some family members who did not stay expressed regret over not being able to perform this expected role:

I sort of, a bit of regret on my part. I did not want to be with [the patient] because I did not want to see her … struggling with her last breath.

(Toby, spouse, OP)

After intense, exhausting periods of decision making and waiting for removal of life sustaining therapies to begin, some family members were unable to remain with the potential donor, despite feeling as though they ought to be. The possibility of witnessing distressing aspects (i.e., respiratory distress) of technologically orchestrated deaths also deterred some participants from remaining at the bedside. Staying with the dying is an important component of the bedside vigil (Caswell et al. 2022; Kellehear 2013) and appears to be a behaviour more common in families of DCC donors than those of brain dead donors (Prescott et al. 2019). The work of the bedside vigil includes protecting the dying person and ensuring they are comforted in their final moments (Kellehear 2013). This work may be more difficult in the technology‐intensive contexts surrounding attempts for DCC donation, where routine processes such as removal of breathing tubes and drawing blood samples to ensure transplant compatibility can be perceived to cause distress. On the other hand, the hospital setting allows family members to share caregiving roles with healthcare providers, permitting respite when the emotional and physical toll of end‐of‐life caregiving becomes too great. In this study, despite whether family members were able to remain at the bedside themselves, the care work of ‘being with’ the patient during the dying process was identified as important.

#### 4.2.3 Anticipation of Death in Time to Donate: ‘Watching the Clock’

Thirty‐one interviews involved cases where patients went through the process of removal of life sustaining therapies with the understanding that if death occurred within 2 hours the patient would proceed to organ retrieval surgery. The experience of anticipating death within this time limit was a prominent theme.

Donna, the spouse of a patient from whom organs were not procured described this experience of waiting for death to occur as positive. In her case, time was spent sharing memories about the potential donor with the healthcare team. Like family members who felt the delay in ventilator removal was a positive extension of the dying period, Donna experienced time waiting for death to occur as ‘more time’ with the patient and an opportunity to make dying meaningful.

In the remaining interviews, participants described the experience of waiting and hoping for death to occur within the 2‐hour time limit using ambivalent or negative language. The waiting period was described as ‘intense’ (Avery, parent, OP), ‘bittersweet’ (Wren, sibling, NP), ‘bizarre’ (Remy, sibling, NP), ‘wild’ (Sawyer, spouse, NP), ‘awful’ (Jamie and Val, parents, NP) and in two separate instances as ‘torture’ (Sawyer, spouse, NP; Theresa, spouse, NP). More than one participant referred to a feeling of ‘watching the clock’ (Alex, parent, OP; Remy, sibling, NP; Jamie and Val, parents, NP). Participants reported having to contend with mixed emotions as their hopes for death to occur swiftly so the donation attempt could succeed seemed to fit uncomfortably with what they perceived as expected behaviours and feelings about death. Wanting to meet deceased donation time limits meant family members were hoping for the patient's death when they had been hoping for survival just a short time before.

… it was like it was a bit surreal, because like on the one hand, it was a little bizarre, you are watching the clock a little bit and feeling like there's only so much time and if we can help people, there's only two hours for this to happen, it was a weird, it was weird, I was prepared in a way for my brother to die because that was what needed to happen for the next step to be able to harvest organs and pass them on to someone who was living and could use them […] so it was this weird feeling of watching the clock and feeling like okay, is this the right thing to do, this is supposed to happen, he needs to let go, we are going to need to let go, and it has to happen in like two hours, it was very, very strange because we wanted to help other people but it also was our brother dying.

(Remy, sibling, NP)

Remy's recurrent use of descriptors such as ‘strange’, ‘weird’ and ‘bizarre’ serve to underscore how they felt their experience was unusual compared to dominant discourses about the dying process. Instead of peacefully attending a dying sibling in his last moments, Remy describes an anxious feeling of watching the clock and hoping for death to happen so someone else could live. As Cheryl explains, hoping for the death of a loved one is not ‘normal’:

… this is where it gets kind of wearing. And you're sort of now praying for someone to pass instead of going through the normal, you know, losing somebody thing. It just sort of plays on your emotions, for sure—for sure.

(Cheryl, spouse, NP)

In addition to the stress of waiting for death to occur within the timelines for organ donation following DCC, participants had to confront uncomfortable feelings that what they were experiencing was outside of expected emotional experiences around dying. In some interviews, family members described wondering whether anything could be done to speed up the process of dying. Many reflected how admitting to wishing for the patient's death was ‘awful to say’ (Rowan, sibling, NP) and was difficult to process, leading to guilt which persisted in the months after death (Jamie, parent, NP). The process of deceased donation after DCC focuses attention on death within 2 hours as a desirable outcome, conflicting with ‘good death’ narratives in which dying is a meaningful period of saying goodbye and death occurs ‘naturally’, on its own terms (Cottrell and Duggleby 2016). This shift in focus on the end point of death rather than the value of the dying period was perceived by some family members as ‘not normal’, leading to emotional discomfort. Processing this discomfort becomes part of the work that family members do to participate in deceased donation.

### 4.3 Reactions to Donation Attempt

Most participants in this study attended patients who attempted donation after DCC. Family members of patients from whom organs or tissues were procured appeared to derive meaning from their loss by thinking about how the donation helped others. In contrast, family members who experienced an unsuccessful donation attempt described feelings of ambivalence, disappointment and frustration. The emotion work of reconciling positive, negative or ambivalent feelings about donation with what was felt to be expected formed another component of donation care work.

#### 4.3.1 Reactions to Successful Donation Attempt: ‘You Have to Feel Good … When Things Helped’

Most family members of patients from whom organs were procured reported positive feelings towards the donation, describing it as ‘the only positive thing in a really awful situation’ (Alex, parent, OP). Harper, the sibling of a DCC donor, described this feeling in the imperative, saying, ‘Well, you have to feel good, when you know that things helped’. In their case, supporting the decision for their sibling to be a donor resulted in organs going to two people at the same hospital.

Some family members suggested donating organs did not make death easier to accept. Nevertheless, they emphasised the positive value of altruism. Theresa, spouse of a patient from whom organs were not procured, expressed ambivalence about her partner's tissue donation:

[The donation] did not make me feel—it did not make [the patient's death] any easier or it did not make it any … Well, no, actually. Maybe a little better […] The fact that somebody may be seeing because of [the patient] makes me feel good. And the tissue may help a burn victim or whomever. That makes me feel good. But everything else is overwhelming that. […] For now, it's still very hard.

(Theresa, spouse, UD)

Narratives of making a positive contribution and ‘helping others’ were important aspects through which participants interpreted death as meaningful. However, as Carol explained, altruism did not erase grief:

I feel a little bit weird about the way I feel, actually, because I was glad that [the patient] was able to donate, but I didn't really draw any significance from his death out of it […] For me, it was the same result. He was still dead.

(Carol, girlfriend, OP)

While the act of donating organs was seen as meaningful and important for all participants, the fact that organs were procured did not necessarily help to make sense of death. The discourse of organ donation as a gift of life offers family members a narrative of benevolence in place of the traumatic circumstances often surrounding death. Nevertheless, many family members continued to experience profound grief.

#### 4.3.2 Reactions to Donation Attempt Where Organs Not Procured: ‘This Huge Disappointment’

Seventeen interviews involved discussions about attempts at donation after DCC in which organs were not procured due to deaths after 2 hours. In most cases, disappointment was framed in general terms that ‘someone couldn't benefit’ (Casey, sibling, NP). Instead of anger or relief at having more time with the body (as patients were not rushed to the operating room for organ retrieval), most family members described feeling sad.

Participating in the donation process built up expectations which were ultimately let down when organs could not be retrieved. Family members reported feeling upset when they realized waiting recipients would not be able to obtain lifesaving organs. The denial of opportunity to these imagined others was experienced as an additional loss. Theresa and Sawyer, both spouses of attempted donors from whom organs were not procured, framed the inability to donate organs as a ‘waste’. Loss of perceived value extended at times to the patient and family, with a few participants reporting feeling as though healthcare providers had less interest and concern in the patient once organ procurement was no longer possible. Our findings mirror previously reported accounts of unsuccessful donation after DCC as a second or third loss for families (Sarti et al. 2025; Taylor et al. 2017).

The possibility of organ donation positions both patient and family members as generous givers of life and offers a positive narrative in a tragic moment. When death does not occur within the required time window, families lose access to this narrative of transformation. Karen, parent of a potential donor from whom organs were not procured, described how not being able to donate organs led to an intensification of her grief.

I remember thinking at about 20 minutes before the two‐hour timeframe, thinking, ‘no, no, no, don't, look, please let these monitors go silent,’ and it was just like, I was beginning to have this, not an anxiousness, but this huge disappointment, and I remember when the timeframe came, how could this be? that was my first reaction, how could this be? That this young man whose heart stops, who was VSA [vital signs absent], and is revived, and here we are a week later, and his heart is still beating strong, like to me it was such a, it was like it didn't make sense, and it was just like, a huge, huge disappointment, I think I probably felt more empty then, than when I heard the news that, you know, there was really no hope of his recovery, because I kept thinking that, that, this, this good would come out of his passing, so for me it was huge, probably one of my lowest moments…

(Karen, parent, NP)

For Karen, the fact that her son was medically revived from a state of death only to not die on time to donate organs was incomprehensible. That death could be successfully controlled in one circumstance but not another seemed nonsensical. Karen's realization that her son would not be an organ donor implied the loss of an imagined future in which ‘good would come out of his passing’. As part of the work of coming to terms with her loss, Karen had constructed a future in which her son became a donor. When death took longer than 2 hours, that imagined future was stripped away, leaving an emptiness worse than death. Dealing with this ‘second loss’ amplifies the work involved in usual end of life care. It is a unique feature of the care work that family members do during deceased donation.

Though some participants mentioned feeling grateful for the opportunity to attempt donation, they did not appear to attribute significant meaning to the experience when organs were not procured—there is no culturally celebrated narrative for the attempt to donate in absence of transplantable organ outcomes.

## 5 Discussion

Interviews with Canadian family members about experiences attending potential DCC donors illustrate ongoing contributions of family members beyond the moment of consent. In this study, family members described how they waited for withdrawal of life sustaining therapies, were present at the bedside during ventilator removal and remained present with patients while waiting for death to occur. Throughout this period, family members also described managing sometimes conflicting emotions about death and possibilities of hope arising from deceased donation. In line with feminist concepts of care work, we argue that these contributions can be understood as work. Family members perform the physical and emotional work of being present and controlling affective responses to death, dying, and organ donation. Over and above the tasks of managing dying, donation care work involves the emotional work of reconciling the impacts of deceased donation on the dying process, including managing difficult feelings arising from wanting death to occur while mourning unexpected loss. Rather than a ‘natural’ or expected response, we argue that donation care work is a particular form of labour that is essential to sustaining the project of deceased organ donation.

Though less obvious than the death‐brokering activities of healthcare workers (Cooper and Murphy 2025), the donation care work performed by family members is essential to the sustainability of deceased donation after DCC. Instances in which family members refuse or are unable to continue this work threaten the possibility of donation. Our study of 36 families included one instance in which a family member threatened to withdraw consent for donation if end of life processes did not proceed in a timely manner. This finding is not unusual—a review of 148 cases of potential donation after DCC indicated the main reasons for family refusal of consent were inability to continue waiting for death and not wanting to see the patient suffer (Verble et al. 2020). The process of deceased donation after DCC requires physically and emotionally exhausting work from family members—work that not all families are willing or able to perform. The fact that many family members do take on this additional labour helps to sustain the project of deceased organ donation.

Emotional aspects of donation care work may also be vital to sustaining broader discourses about donation that help to foster public trust. Some family members in this study mentioned having wished for their loved one's death to achieve donation, a thought which was described as a ‘horrible thing to live through’ (Jaime, parent, UD). Although a ‘good death’ may be perceived by some as rapid and painless (Selter et al. 2022), our findings suggest hoping for rapid death for the patient's sake may be experienced differently than hoping for death to achieve donation. The perceived shift from a focus on the patient's wellbeing to the transplantable organs derived from death sits awkwardly with some family members and appeared to create psychological dissonance. Disjunctions between a discourse of meaningful dying and experiences of deceased donation resulted in some family members feeling ‘weird about the way [they] feel’ (Carol, girlfriend, OD), even, in Carol's case, when organ procurement did occur. Family members' willingness to do the emotional work of adapting uncomfortable feelings that death did not occur as expected facilitate perpetuation of dominant narratives of gratitude and altruism. Donation care work involves making sense of conflicting feelings in such a way as to permit the larger collective social enterprise of deceased donation to persist. Its performance by family members is therefore an integral component of what Bea (2020) names as the larger ‘assemblage’ of donation after DCC.

Framing family contributions as work also helps to foreground potential inequalities and imbalances of power. In our study, most family member participants were women performing donation care work on behalf of male partners or children. This demographic distribution aligns with other empirical studies of donor families (Renet et al. 2026; Sarti et al. 2022, 2025) and indicates a trend towards gender imbalance in who performs the work of donation care. Although some overrepresentation of women family members may be explained by recruitment and participation patterns of qualitative bereavement research (Stroebe et al. 2003), it also reflects the reality that men are more likely to die in contexts in which deceased donation is possible and are likely to have female partners or caregivers (Rogers 2022). In response to these patterns, donor family support initiatives should consider how gender roles and expectations shape experiences of donation care work. This is an important task for future study.

Understanding contributions of family members as labour introduces a new lens for thinking about the sociology of donation. The concept of donation care work draws attention to the emotional and physical demands that deceased donation after DCC makes on family members. Sociological and legalistic frameworks of donation have tended to view family members as gatekeepers to the individualised act of donation while obscuring the work required to sustain donation processes once consent is obtained (Toews and Caulfield 2016). Family refusal of donation is positioned as a failure due to lack of knowledge or cultural attitudes, and potentially as an illegal act when it contravenes prior stated wishes (D. Shaw et al. 2017; Verble et al. 2020). The concept of donation care work highlights the commitments produced through the act of giving, complementing prior work by Fox and Swazey (1992) on obligations generated through receiving a donated organ. By registering as donors, individuals may implicitly enlist family members into the practical and emotional labour required to realize that gift after death. Understanding deceased donation as relying on family member care work adds families' capacity to perform this work as an important dimension of donation decision‐making. Our proposed concept of donation care work reorients attention from family members as gatekeepers of consent towards their role as essential contributors to the donation process, whose labour constitutes a condition of possibility for deceased donation.

Our work positions family members as playing an active role in deceased organ donation and contributes to theoretical conceptions of deceased organ donation as a collective social effort, rather than an individualised gift. For example, Łuków (2020) and Zeiler (2014) theorise donation as a collective effort made possible through an ethics of shared struggle and suffering among multiple actors. Moving away from donation as an individual gift foregrounds contributions involved throughout the entire donation attempt, from discussions around consent to the moment of death determination and into the bereavement period that follows. This pivot is essential for understanding social meanings of donation in the context of DCC. Donation after DCC is socially meaningful because of the donation care work family members do to make attempts possible, even when desired outcomes of transplantable organs do not result. Contributions of time and emotional fortitude deserve recognition as work which maintains the donation system. Developing a cultural narrative in which collective act rather than individual outcome of donation is celebrated may help to better reflect the realities of the experience.

## 6 Conclusion

This study of family members' experiences with donation after DCC illustrates how activities of waiting for death to occur and processing difficult, conflicting emotions can be understood as labour that helps to make the process of deceased donation possible. To our knowledge, this is the first study to theorise family experiences of deceased donation attempts as care work, positioning these activities as essential to sustaining the larger collective enterprise of deceased organ donation. By extending feminist theories of care to deceased organ donation, the concept of donation care work shifts attention from consent decisions to the emotional, relational and temporal labour that sustains the social infrastructure of deceased donation. Our findings have implications for how donor families are understood, supported and valued in organ donation systems while also contributing to sociological analyses of how unpaid family labour sustains healthcare institutions.

## Author Contributions

**Amanda van Beinum:** conceptualization, validation, formal analysis, investigation, data curation, writing – original draft, writing – review and editing, project administration, methodology. **Vanessa Gruben:** conceptualization, methodology, investigation, writing – review and editing, data curation, project administration, formal analysis, validation. **Aimee J. Sarti:** formal analysis, validation, data curation, writing – review and editing. **Sonny Dhanani:** resources, funding acquisition, writing – review and editing, supervision. **Lindsey McKay:** conceptualization, validation, formal analysis, investigation, data curation, writing – review and editing, methodology. **Jennifer A. Chandler:** conceptualization, formal analysis, data curation, funding acquisition, investigation, methodology, validation, writing – review and editing, project administration, supervision.

## Funding

This work was partially funded by the Bertram Loeb Research Chair and by the Canadian Institutes for Health Research as part of the Canadian Donation and Transplantation Research Program. No specific funding was granted for the preparation of this article.

## Ethics Statement

This study was approved by the local research ethics board (File no. 04‐15‐25, File no. 04‐15‐25B), the research ethics boards of all participating hospitals, and the research committee of the organ donation organisation.

## Consent

Written consent was obtained from all participants in this study prior to each interview.

## Conflicts of Interest

Sonny Dhanani is a Hospital Donation Physician and holds positions with Ontario Health and the Trillium Gift of Life Network. Jennifer A. Chandler previously held the Bertram Loeb Chair in Organ and Tissue Donation. All other authors declare no conflicts of interest.

## Supporting information


Supporting Information S1

## Data Availability

The participants of this study did not give written consent for their interview transcripts to be shared publicly. Due to the sensitive nature of the research, supporting data beyond the anonymized quotes contained in this manuscript are not available.

## Endnotes

^1^
Withdrawal of life sustaining therapies is a controlled medical process for removing organ support therapies to allow death to occur. It generally involves a reduction of medications used to support the heart and circulatory system and a removal of artificial ventilation while administering drugs for pain relief and sedation to control any discomfort (Downar et al. 2016).

^2^
Canadian donation and transplantation programs are developed and run by each province and territory. At the time of data collection, all provinces and territories operated under opt‐in models in which people must register their intent to donate, and families are asked for confirmation of consent. As of 2021, Nova Scotia passed legislation to move to an opt‐out system in which consent is presumed unless a person has registered not to donate (Manitoba Law Reform Commission 2022).
