# Supplementary material for: Donation Care Work: Family Members' Contributions to Deceased Organ Donation Following Determination of Death Using Circulatory Criteria
Source: Sociol Health Illn. 2026 Sep 6;48(7):e70257. doi: 10.1111/1467-9566.70257 (PMC13547768; doi:10.1111/1467-9566.70257)
Supplement: Supplementary file 1 [file SHIL-48-0-s001.pdf]

INTERVIEW GUIDE

QUESTION 1 – OPEN-ENDED

Ask interviewee to take us through the story of what happened, in their own words.

[Interviewers should take notes of things to explore further in follow up questions]

General Probes if needed:

- What happened when you got to the hospital?
  - Who was with you?
  - Where were you?
  - Staff: Who spoke to you?
  - Did you get the information you wanted and needed?
  - Would you change anything about how you were treated at this time?
- How long was your loved one in hospital?
  - What was the course of her/his condition?
  - Did the prognosis change very much?
- When did you know [person] was gone?
  - What signs of "the end" mattered to you?

QUESTION 2 - DECISION-MAKING – WLST

Can you tell me/us about the timeline of WLST?

- How and when did it happen? who was involved, how much decision-making power was given to families to decide what happened and when

- **How did you come to the decision to [stop treatment]?**
  - Probes if needed
    - What kinds of things did you consider in making this decision?
    - Did you have enough time to make your decision?
    - Were you encouraged or discouraged from stopping treatment in any way? How?
    - Would you make the same decision again? What, if anything, did you think about the life-sustaining treatments?
    - What would you tell other families going through the same thing?

- **Can you tell us <u>what happened after</u> you made the decision [to stop treatment]?**
    - Probes if needed:
        - Were you present for the removal of treatment?
        - What was the timeline of withdrawal of life sustaining treatment like?
            - Did <u>your loved one</u> remain in the same location?
            - Did new healthcare team members appear?
        - Did the doctors and nurses <u>begin removing interventions</u> <u>right away, or was this process more planned</u>? How did you feel about the time in between?
        - <u>Info</u>: How was the process explained to you? Did you want more or less information than you received?
        - <u>Control</u>: Were you able to decide what was removed, and when? Did anyone provide you those options? (e.g. removal of breathing machine, removal of tubes)
- **Can you tell us what happened after <u>your loved one</u> died? (If SDM was present for WLST)**
    - Probes if needed:
        - Did you remain in the room? In the hospital?
        - Did you feel you had enough time?
        - Who was there? What did the healthcare team do?

<[If moving chronologically along the timeline]: Can you recount being asked to participate in the physiological study? Do you remember when you discussed this? I'd like to return to this matter later in the interview (Q6-8]>

QUESTION 3 - DECISION-MAKING – DONATION

- **Can you tell us how you came to the decision to [consent to donation]?**
    - Probes if needed
        - <u>Who</u> raised the possibility of donation?
        - Had you <u>thought about it before</u> it was raised with you?
        - What were you told about donation?
            - Did you get the information you wanted about donation?
        - Did you <u>have enough time</u> to make your decision?
        - Were you <u>encouraged or discouraged</u> to donate in any way, how, by whom?
        - <u>Why</u> did you decide [for][against] donation?

- Would you make the same decision again?

QUESTION 4 – DECISION-MAKING – INTERACTION

[…get at the ethical worry about perceived conflict of interest]

- Looking at your timeline, can your point to when the possibility of donation was raised?
    - Probes:
        - Was it before, after, or at the same time as the discussion about whether to stop treatment and allow your loved one to pass away?
        - Were the decision to withdraw treatment and the decision to donate <u>separate in your mind</u>?
        - Did you think about donation when you were deciding to withdraw treatment?
        - Were you thinking about them both at the same time, or did you think about one first then the other? Which one did you think about first?

- Do you think that the possibility of organ donation <u>affected the medical care</u> for your loved one in any way?
- Did donation have an impact on your feelings about his/her death? (Did it make it easier or harder to let a loved one go, or did it have no effect?)

QUESTION 5 – DECISION-MAKING – EVALUATION OF DONATION

- **Do you know the outcome of the donation [successful][unsuccessful]?**
    - Probes if needed
        - Who told you about the outcome?
        - Were you told that the donation might not be successful?
- **How do you feel about the outcome [successful][unsuccessful]?**
    - Probes if needed
        - Would you make the same decisions again (about WLST and OD)?

++

<u>QUESTION 6 – PARTICIPATION IN STUDY – OPEN-ENDED</u>

- **You participated in a research study at the time that your ________ died, can you tell us about that experience?**
    - Probes if needed:

- Who spoke to you about the study?
- What do you remember about the study?
- Were you given the information you needed?

QUESTION 7 – PARTICIPATION IN STUDY – REASON FOR CONSENT

- **Can you tell us why you consented to the study?**
    - Probes if needed:
        - Do you remember signing a consent form for the research study?
        - Was anyone else involved with you in consenting?

QUESTION 8 – PARTICIPATION IN STUDY – AWARENESS OF MONITORING

- During the study, the researchers recorded things like heartbeat and blood pressure using the existing bedside monitors in the ICU. The study collected data during the process of withdrawal of life sustaining therapies and after the declaration of death.
- How did you feel about this monitoring?
    - Probes if needed:
        - Were you aware that the monitoring was taking place?
        - How did you feel knowing that the study was taking place?
        - Did the study interfere with your ability to be with your loved one at that time?
        - Did your experience match what you'd been told about the study?
        - Do you have any advice for researchers doing studies at such a sensitive and difficult time?

QUESTION 9 – WRAP-UP

- Is there anything else that doctors, hospital staff or researchers should know about your experience?
